# Associations Between Social Functioning and Indicators of University Student Engagement

**DOI:** 10.3390/ejihpe15060099

**Published:** 2025-06-03

**Authors:** Marco Turi, Rocco Servidio, Giovanna Esposito, Flaviana Tenuta, Lorena Montesano, Andrea De Giacomo, Antonella Valenti, Maria Francesca Freda, Linda S. Pagani, Francesco Craig

**Affiliations:** 1 Department of Human and Social Studies, University of Salento, 73100 Lecce, Italy; marco.turi@unisalento.it; 2 Department of Cultures, Education and Society, University of Calabria, 87036 Rende, Italy; rocco.servidio@unical.it (R.S.); flaviana.tenuta@unical.it (F.T.); 3 Department of Humanities, University of Naples Federico II, 80138 Napoli, Italy; giovan.esposito@unina.it (G.E.); fmfreda@unina.it (M.F.F.); 4 Department of Mathematics and Computer Science, University of Calabria, 87036 Rende, Italy; lorena.montesano@unical.it (L.M.); antonella.valenti@unical.it (A.V.); 5 Department of Translational Biomedicine and Neurosciences (DiBraiN), “Aldo Moro” University of Bari, 70100 Bari, Italy; andrea.degiacomo@uniba.it; 6 School of Psycho-Education, University of Montreal, Montreal, QC H3C 3J7, Canada; linda.s.pagani@umontreal.ca

**Keywords:** social functioning, academic engagement, university student, broad autism phenotype, psychological distress

## Abstract

Less socially adaptive behaviors have often been underestimated in university students, with limited research addressing their impact on academic functioning. This study aimed to identify distinct profiles of social functioning difficulties in university students and to examine their associations with academic engagement, learning difficulties, and psychological distress. A cross-sectional, web-based survey was conducted with 540 undergraduate university students (mean age = 23.06, SD = 6.53; 89.7% female). Participants completed standardized self-report assessments of social functioning (SRS-2), academic engagement (SAES), learning difficulties (Vinegrad Plus), and psychological distress (GAD-7, PHQ-9). Latent profile analysis (LPA), based on ASD-related traits, revealed two latent profiles: one reflecting non-social functioning difficulties (311 participants, 57.6%—Profile 1) and another reflecting social functioning difficulties (229 participants, 42.4%—Profile 2), while binomial regression analyses examined their associations with academic outcomes. Participants in Profile 2 scored significantly higher than those in Profile 1 across all SRS-2 variables—awareness, cognition, communication, motivation, and restricted interests and repetitive behavior (*p* = 0.001)—indicating greater overall functioning in these domains. Students in the Non-social functioning difficulties profile showed higher levels of academic engagement in all areas. In contrast, students in the Social functioning difficulties profile experienced more self-reported learning challenges (*p* = 0.001), anxiety (*p* = 0.001), and depression (*p* = 0.001), underscoring the significant differences in social, academic, and emotional outcomes between the two profiles. These findings underscore the impact of vulnerability to social functioning difficulties on academic engagement, highlighting the need for tailored support systems within higher education settings.

## 1. Introduction

Some adults in the general population report or exhibit habitual behavior that would be considered less effective in social interactions. When such social vulnerability patterns are more pronounced, they collectively represent a social functioning that can be conceptualized as a trait. In this regard, the social skills deficit vulnerability model (Curran & Elwood, 2024; Segrin et al., 2016) offers a particularly useful theoretical framework for understanding the links between social communication difficulties and broader psychological outcomes. Specifically, the model suggests that limited social competence increases the risk of negative psychosocial outcomes, such as loneliness, anxiety, and depression. These behavioral patterns may include suboptimal social skills, cognitive rigidity, and apprehensive, avoidant, and/or detached behaviors (Iannuzzo et al., 2022). Although such difficulties may fall within the broader autistic phenotype, it is important to note that a diagnosis of Autism Spectrum Disorder (ASD) is warranted only when these behaviors are pervasive, persistent, and result in significant functional impairment (Gesi et al., 2021). Adults in the general population may exhibit social functioning difficulties, showing specific patterns such as poor social skills, cognitive rigidity, anxiety, and aloofness (Iannuzzo et al., 2022). An expanding body of literature further suggests the presence of autistic trait characteristics along a continuum in the general population, ranging from more to less clinically significant impairments (McLeod & Anderson, 2023). These traits often manifest as difficulties in integrating into social groups and building interpersonal relationships, as well as avoiding social contact (Fusar-Poli et al., 2020; Kentrou et al., 2024; Lai et al., 2017; Yu et al., 2023). However, little is known about the association between social functioning difficulties and student engagement, especially for females, who often camouflage such characteristics within gendered social expectations regarding typically reserved female behavior (Lai et al., 2017).

Although a universally agreed upon definition of social skill vulnerability has yet to be established, the following social functioning difficulties consistently emerge in the literature (Green et al., 2015; Happé et al., 2017; Happé & Frith, 2013; Huber et al., 2018; Pallathra et al., 2018): (a) difficulties in perceiving and interpreting; (b) reduced motivation to engage in social contact; and (c) impairments in the skills required to initiate, maintain, and conclude social interactions. Previous studies have shown that greater difficulties in social functioning among college students are associated with fewer and lower-quality friendships, increased loneliness, and greater internalizing of symptoms such as anxiety and depression (Faso et al., 2015; Mallory & Keehn, 2021; Reed et al., 2016). These findings underscore the need for targeted support for both diagnosed and undiagnosed individuals across various contexts, including educational, occupational, and everyday life settings. However, given the non-clinical nature of the current sample and the use of a screening instrument, we chose to refer to social functioning difficulties rather than autistic traits, to adopt terminology that more accurately reflects the functional variability within a general university population. This approach allowed for a neutral and descriptive account of the observed difficulties, avoiding the use of labels that may imply a neurodevelopmental condition in the absence of a clinical assessment.

Academic engagement is a multidimensional construct, with social, affective, and behavioral dimensions (Fredricks, 2022). These dimensions influence students’ ability to invest in meaningful relationships with peers and instructors, commit to their academic institution, and perceive the university environment as interconnected with other aspects of life (Fredricks, 2022). Indeed, students with strong connections to their institutions, teachers, and peers are more likely achieve academic success and remain on their educational path (Guzzardo et al., 2020; Martinot et al., 2022). Furthermore, Kiema-Junes et al. (2020) found that social skills predict higher engagement and lower burnout among university students, suggesting that enhancing social skills could foster student engagement. Understanding academic engagement is therefore key to mitigating dissatisfaction, preventing boredom, enhancing motivation, and improving academic performance (Freda et al., 2021). Greater insight into engagement in the university population is warranted to enrich the educational experience—particularly for students displaying psychosocial vulnerabilities (Martinot et al., 2022).

Support services primarily target students with documented medical diagnoses, which is less inclusive. Addressing social functioning dimensions, such as social cognition, motivation, and communication, across the entire student population could help to tailor interventions and elucidate the underlying mechanisms of autism spectrum disorders. Furthermore, understanding the relationship between social skill deficit vulnerability and indicators of academic engagement could influence the future implementation of academic support services.

Cluster analysis has been employed in previous studies to explore social phenotypic heterogeneity within student populations. Palmer et al. (2015) demonstrated that a large sample of university students (n = 2.343) could be divided into two clusters based on responses to the Autism Spectrum Quotient (AQ) (Baron-Cohen et al., 2001). Their findings identified two main subtypes, with one group showing pronounced social difficulties and low detail orientation, and the other displaying milder social challenges alongside a stronger focus on detail. This supports a dimensional, continuum-based view of autistic traits over categorical models. Similarly, James et al. (2016) investigated the underlying structure of autistic traits measured through the AQ among university students and adults, employing latent class and profile analyses. Their results suggested a categorical structure of autistic traits, indicating that such traits may cluster into distinct profiles, rather than existing solely along a continuous dimension.

Latent profile analysis is an analytical approach used to categorize individuals into subgroups based on a set of observed variables, with the goal of capturing heterogeneity either qualitatively or quantitatively (Tillmann et al., 2020). Broadly speaking, person-centered methods allow for the empirical identification of subgroups of individuals who share similar or dissimilar characteristics, whereas variable-centered methods—such as clustering techniques—are designed to examine general relationships among variables across the entire population (Bauer et al., 2022).

The objective of this cross-sectional study was to adopt a person-centered approach to identify vulnerabilities in social skills among university students and to evaluate the extent to which these deficits are linearly associated with academic outcomes. Specifically, the study sought to identify distinct profiles of social functioning and examine how these profiles relate to various indicators of academic engagement. Generating distinct profiles within a university population facilitates a shift from a generalized to a more individualized perspective. It was hypothesized that students exhibiting social functioning difficulties would report lower levels of academic engagement compared to their peers with more adaptive social functioning profiles. The findings have the potential to inform the development of targeted strategies to enhance engagement among students with social functioning challenges, thereby promoting more favorable academic outcomes.

## 2. Materials and Methods

### 2.1. Participants

The current study protocol was approved by the Institutional Review Board (IRB) (protocol number: n. 0533634—October 2023) and conducted in accordance with the principles outlined in the Declaration of Helsinki. Participants were university students aged 18 years or older enrolled at the University of Calabria.

The initial sample included 553 students. An open-ended question in the survey assessed whether participants had any prior diagnosis of neurodevelopmental disorders. Thirteen participants were excluded as they reported having a certified diagnosis that impeded their ability to complete the tests. The final sample consisted of 540 students, with a mean age of 22.49 years (SD = 5.15). Among them, only 54 were male (mean age = 23.06 years, SD = 6.53; age range = 18–58 years), and 471 were female (mean age = 22.41 years, SD = 4.89; age range = 18–51 years). Regarding academic areas, 227 students were enrolled in Education programs, 62 in Medicine and health programs, and 80 in Engineering and technology disciplines. A total of 171 participants did not report their academic field of study, leaving this optional question blank.

### 2.2. Procedures

Convenient response sampling was used to collect data through questionnaire links from November 2023 to May 2024. Participants were invited to take part in the online survey by completing Google Forms. No incentives were provided to the participants. The data collected through the online questionnaire were completely anonymous, and the responses were used solely for statistical purposes, in compliance with the European Data Protection Regulation GDPR 679/2016 and to safeguard privacy. Written informed consent was obtained from all enrolled participants. To minimize the risk of duplicate responses on the questionnaires, each participant was restricted to accessing the survey solely through their university identification code, limiting them to one response.

The online survey was organized into four main sections: (1) informed consent and data handling; (2) socio-demographic information, including age, biological sex, residential status (university residence, living with family, apartment, apartment with roommates, or other arrangements), type of degree program, and year of university enrollment; (3) screening and clinical questionnaires, which comprised the Social Responsiveness Scale-Second Edition (SRS-2), the Vinegrad Plus, the Patient Health Questionnaire (PHQ-9), and General Anxiety Disorder (GAD-7); (4) academic engagement, measured using the SInAPSi Academic Engagement Scale (SAES).

The survey was distributed to students via a link or QR code during in-class sessions. One of the investigators provided an orientation and guided participants through the completion of the questionnaire.

#### 2.2.1. Measures—Predictor Variable: Social Functioning

To identify and quantify social impairment, we used the SRS-2 (Constantino & Gruber, 2012), a highly regarded autism assessment that offers the convenience of a screener and the power of a diagnostic tool. The authors of the questionnaire suggest that, because scores along a continuum can be obtained, the SRS-2 can help clinicians identify and understand people on the autism spectrum with milder impairments, as well as individuals with non-ASD conditions who also show social impairments. SRS-2 scores are categorized as T = 59 or lower indicating typical functioning, T = 60–65 representing mild impairment, T = 66–75 indicating moderate impairment, and T = 76 or higher indicating severe impairment. Completed in just 15 to 20 min, the SRS-2 identifies social impairment associated with ASDs and quantifies its severity. Individuals are asked to rate 65 items about their child’s behavior over the past six months using a Likert scale from 1 (not true) to 4 (almost always true). High scores are associated with more severe social impairments. Items cluster into five subdomains that correspond to the overarching two-factor structure of DSM-5 diagnostic domains—Social Communication and Interaction (Social Awareness, Social Cognition, Social Communication, and Social Motivation subdomains) and Restricted, Repetitive Behaviors and Interests (Constantino & Gruber, 2012). The SRS-2 has strong psychometric properties in clinical (ASD) and non-clinical standardization samples. The internal consistency reliability is α = 0.95 and 0.97, respectively. In the present study, the SRS-2 demonstrated good internal consistency, with a Cronbach’s alpha coefficient of 0.86. Given that the SRS-2 is designed to identify distinct profiles of social functioning, the internal consistency was calculated separately for each subscale using Cronbach’s alpha. The resulting coefficients were as follows: Social Awareness (α = 0.82), Social Cognition (α = 0.78), Social Communication (α = 0.80), Social Motivation (α = 0.66), and Restricted Interests and Repetitive Behavior (α = 0.62). These values indicate good reliability for the first three subscales, while the latter two demonstrate acceptable to marginal reliability, suggesting that findings related to these domains should be interpreted with caution.

#### 2.2.2. Measures—Outcome Variables: Indicators of Academic Engagement

The SInAPSi Academic Engagement Scale (Freda et al., 2021; Passeggia et al., 2023) was developed and validated for university engagement. This 29-item self-report scale is structured on six dimensions: (1) Perception of the capability to persist in the university choice (e.g., I’d leave the university right away if I had an alternative); (2) University value and sense of belonging (e.g., Attending university is a great opportunity for me); (3) Value of university program (e.g., I find my studies very significant for my professional plans); (4) Relationships between university and personal network (e.g., I talk about my professional plans with my family); (5) Engagement with university peers (e.g., Studying with other students is useful to me); (6) Engagement with university professors (e.g., My instructors are interested in my opinions and what I say). Responses are on a five-point Likert-type scale ranging between 1 (not at all) and 5 (definitely). A higher score in the SAES indicates an excellent level of university engagement. The internal consistency of the SAES is α = 0.95. In our study, the internal consistency of the instrument, as assessed by Cronbach’s alpha, was 0.92, indicating excellent reliability.

#### 2.2.3. Measures—Outcome Variables: Perception of Emotional Distress and Learning Difficulties

The GAD-7 (Spitzer et al., 2006) is a 7-item self-report measure of the severity of symptoms related to generalized anxiety disorder in adult individuals. Questions are structured to inquire how often the subject has experienced specific symptoms over the past two weeks. Symptoms assessed include excessive worry, difficulty controlling worry, restlessness, nervousness, feeling tense or irritable, and difficulty relaxing. Responses are in multiple-choice format on a four-point scale based on the frequency of experiencing the symptom, from 0 (not at all) to 3 (nearly every day). Total scores range from 0 to 21, where higher scores indicate greater severity of anxiety. The GAD-7 is widely used in clinical and research settings to assess the presence and level of generalized anxiety, as well as to monitor treatment response over time. It is an effective and time-efficient tool meant to help identify patients who may benefit from anxiety treatment. The internal consistency of this instrument is α = 0.92. The instrument showed good reliability in our study, with a Cronbach’s alpha of 0.86, reflecting strong internal consistency.

The PHQ-9 (Spitzer et al., 1999) is a 9-item self-assessment tool commonly used in clinical settings to evaluate depressive symptoms in adults. These include feelings of sadness, worthlessness, and guilt; lack of motivation; loss of interest or pleasure in activities; changes in sleep or appetite; fatigue or lack of energy; and difficulty concentrating or making decisions. The PHQ-9 serves as an important tool in clinical psychology and psychiatry for evaluating the mental health status of patients. It is widely utilized by mental health professionals to aid in screening for depression, monitoring symptom changes over time, and assessing treatment response. Item responses are on a four-point scale based on the frequency of experiencing the symptom over the past two weeks from 0 (not at all) to 3 (nearly every day). Total scores range from 0 to 27, with higher scores indicating greater severity of depressive symptoms. The internal consistency of this instrument is α = 0.89. The internal consistency of the instrument was found to be satisfactory in the current study, as indicated by a Cronbach’s alpha coefficient of 0.84.

The Vinegrad Plus Self-Assessment Questionnaire is a 26-item self-report scale designed to assess self-perceived current difficulties related to learning. It focuses on abilities in daily tasks involving reading and writing, difficulties in numeracy, and challenges related to automatisable aspects of social skills and language. Responses are dichotomous, with a “yes” or “no” format. This questionnaire is part of the Lettura Scrittura Calcolo—Studenti Universitari e Adulti (LSC-SUA) battery (Montesano et al., 2020). In the present study, the Cronbach’s alpha was 0.82, indicating good internal consistency and reliability of the instrument.

### 2.3. Analysis

The data were analyzed using the R environment and Jamovi version 2.3.28 (Lino Calle et al., 2024). Descriptive statistics were determined to be normally distributed for all key study variables.

To address the study aims, LPA was conducted using the five subscales of the SRS-2 as continuous indicators: Social Awareness, Social Cognition, Social Communication, Social Motivation, and Restricted, Repetitive Behaviors and Interests. This analysis was conducted using the TidyLPA package in R (Rosenberg et al., 2018). The LPA method, known for its data-driven modelling approach, helps identify naturally occurring homogeneous subgroups (profiles) within a population based on meaningful descriptors or distinctive characteristics; in this case, the scale determined the absence or presence and severity of social impairment within autism spectrum disorder. TidyLPA was selected for its capacity to predict optimal relationships between indicators across different profiles, including means (e.g., average levels of absence or presence and severity of social impairment), variances (e.g., variability of presence and severity of social impairment within profiles), and covariances (e.g., variability of presence and severity of social impairment scores across identified profiles). This package enables the assessment of four different model parameterizations (Masyn, 2013). It is worth noting that there is no minimum sample size requirement for LPA. However, simulation studies suggest a threshold of over 500 participants is advisable, with more informative indicators potentially compensating for smaller sample sizes (Footitt et al., 2024; Kongsted & Nielsen, 2017). Both these recommendations were met for the analyses.

Specifically, the process of determining the optimal number and structure/parameterization of latent profiles involved the following steps. Initially, we identified the best combination of parameters (including non, partially, and fully constrained profile means, variances, and covariances) by comparing models based on various fit criteria. Next, the optimal number of profiles was evaluated using the bootstrapped likelihood ratio test (BLRT). The BLRT assesses whether adding a latent profile significantly improves the model fit, with *p* < 0.05 indicating a better fit (McLachlan, 1987). If the BLRT is non-significant, adding another profile does not improve the model fit. Finally, we assessed the heterogeneity levels across latent profiles by examining the standardized entropy index (Wang et al., 2017).

After identifying the most parsimonious latent profile solution using LPA, we categorized participants into two distinct profiles based on their SRS-2 scores and subsequently examined group differences across both categorical and continuous variables. For categorical variables, chi-square tests of independence were used to detect significant differences in distribution across profiles. For continuous variables, we employed independent samples t-tests and one-way ANOVA. These statistical tests provided complementary evidence regarding the distinguishing characteristics of each profile and facilitated a richer interpretation of the latent classes.

After analyzing group differences, we conducted a hierarchical logistic regression to examine the extent to which the external variables predicted profile membership, as identified through latent profile analysis (LPA), with Profile 1 coded as the reference group. The analysis was structured using a three-block hierarchical approach, allowing us to assess the incremental predictive value of conceptually distinct sets of variables. Block 1 included demographic variables (age and sex), which are commonly associated with social functioning and may influence profile membership. Block 2 introduced distress variables (anxiety and depression) to evaluate their additional contribution beyond demographics. Finally, block 3 included psychosocial factors or behavioral measures (academic difficulty and academic engagement), hypothesized to be most proximally related to differences between profiles. Each block was entered sequentially, and model fit improvements were assessed at each step. This approach allowed us to isolate the unique predictive power of each set of variables, while controlling for those previously entered. Odds ratios (ORs), 95% confidence intervals (CIs), and associated *p*-values were reported for each predictor, to indicate the strength and direction of effects.

## 3. Results

Based on the total T-scores derived from the SRS-2, the sample exhibited the following distribution of social functioning impairment levels: 57.6% demonstrated no impairment, 22.2% showed mild impairment, 16.3% exhibited moderate impairment, and 3.5% were classified as having severe impairment. Regarding other demographic characteristics, most participants were enrolled in the first academic year (176; 32.6%) or the third academic year (289; 53.5%). Most participants reported living in a university residence (270; 50%) or with their parents (179; 33.1%). Finally, most respondents were enrolled in an education degree program (452; 83.7%).

### 3.1. Latent Profile Identification

**Figure 1:**
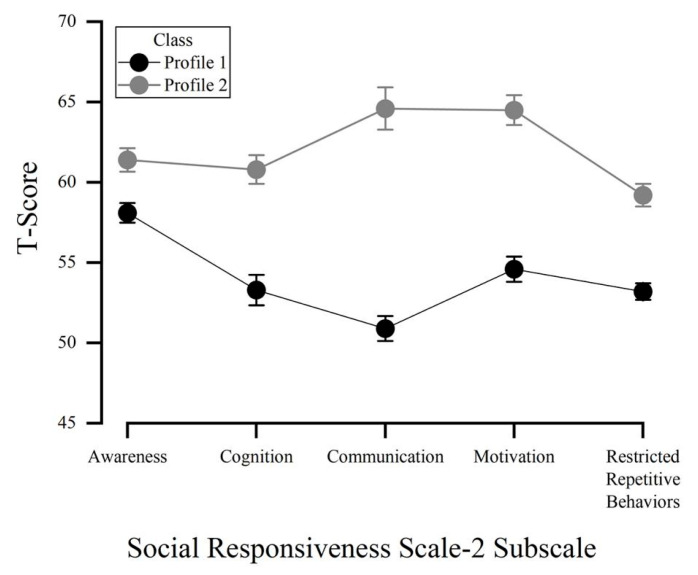
SRS-2 latent profiles. Profile 1: “Non-social functioning difficulties”; Profile 2: “Social functioning difficulties”. Error bars represent S.E.M.

A comprehensive description of the model selection process is available in Appendix A (see Table A1 and Table A2). We characterized the two latent profiles as “Non-social functioning difficulties” (Profile 1) and “Social functioning difficulties” (Profile 2). The proportion of participants in each estimated profile was n = 311 (57.6%) for profile 1, and n = 229 (42.41%) for Profile 2. It should be noted that the smallest class proportion and number exceeded 5% and/or N > 50, as recommended by O’Donnell et al. (2017). The two latent profiles had differences in the scores and average values for the five subscales of the SRS-2. Figure 1 illustrates the mean differences in SRS-2 characteristics across the identified latent profiles.

### 3.2. Differences Between Profiles and Regression Analysis

Table 1 reports the main properties of the two latent profiles according to sex and age. We did not find any significant differences between the two latent profiles for sex, χ^2^(1) = 0.54, *p* = 0.76. On the contrary, a significant age difference was found, tWelch(505) = 3.15, *p* < 0.01. The effect size, as measured by Cohen’s d, was 0.26, indicating a small effect. The results of the ANOVA suggested that all the differences in the SRS-2 scores were significant (all *p*-value < 0.05) according to the two latent profiles (see Table 2). Profile 1 also demonstrated higher academic engagement across all dimensions. In contrast, Profile 2 had greater self-perceived learning difficulties (*p* = 0.001), anxiety (*p* = 0.001), and depression (*p* = 0.001), highlighting distinct differences in social, academic, and emotional characteristics between the profiles.

Hierarchical logistic regression (Table 3) was performed to examine the association between age, sex, learning difficulties, anxiety, and depression symptoms for the two latent profiles on the odds that participants had higher scores for social impairment (reported in Table 3). The binomial logistic regression model was statistically significant, χ^2^(11) = 184.68, *p* < 0.001. The model explained between 29% (Cox Snell R^2^) and 39% (Nagelkerke R^2^) of the variance in mild to moderate impairment and correctly classified 73% of cases of normal functioning. In our sample, age was a significant predictor (*p* = 0.017) of outcome, with older individuals being less likely to have higher scores for social impairment. Psychological variables were important contributors, with higher anxiety (*p* = 0.005) and depression (*p* = 0.001) scores increasing the odds of having higher scores for social impairment. Belonging to the “Social functioning difficulties” profile predicted odds of diminished academic engagement, such as perceived ability to persist in their choice to attend university (*p* = 0.007), sense of university value and belonging (*p* = 0.042), relationships between university and personal networks (*p* = 0.002), and engagement with university peers (*p* = 0.031).

## 4. Discussion

### 4.1. Social Functioning Profiles

This study aimed to identify latent vulnerability to difficulties with social functioning in a university student population. By identifying latent profiles, the study provides a more nuanced understanding of individual differences in social skills beyond traditional categorical approaches, potentially helping to detect at-risk groups who could benefit from early support and targeted interventions. Two profiles were identified: one where more than half of the students reported few or no social interaction or communication difficulties, and another where less than half reported difficulties with social functioning. The current findings differ from those of Farahi and Leth-Steensen (2022), who used similar analyses with over a thousand university participants. They identified four latent profiles for autistic traits. The discrepancies between our study and the previous research may be attributed to several factors, such as sample size and the use of different assessment tools. Specifically, their study utilized the AQ (Baron-Cohen et al., 2001), while our research employed SRS-2. The AQ assesses autism spectrum traits in the general population, focusing not only on social skills and communication, but also on cognitive dimensions such as attention to detail, attention switching, and imagination. In contrast, the SRS-2 is a more specialized tool that evaluates the dimensions of social behaviors. Unlike the AQ, which is broader in scope, the SRS-2 delves deeply into social aspects of behavior, making it particularly useful for identifying social difficulties in non-clinical contexts.

Another possible explanation for the differences in the identified profiles may lie in the sample characteristics. Notably, our sample consisted predominantly of female students, a demographic factor that could have influenced the observed patterns of social functioning. Prior research has suggested that females may present autism-related traits differently, often exhibiting better compensatory strategies in social contexts (Lai et al., 2015). This could result in underreporting or subtler manifestations of social difficulties, potentially affecting the detection and categorization of latent profiles. Thus, the sex distribution in our sample might have contributed to the emergence of fewer and broader profiles compared to studies with more gender-balanced or male-dominated samples. Future research could benefit from exploring gender-specific manifestations of social difficulties using larger and more heterogeneous samples. Moreover, our results indicated that younger students were more likely to fall into the social functioning difficulties profile, a finding that may reflect the unique psychosocial challenges associated with the early stages of university life. Entering higher education often involves navigating new academic pressures, unfamiliar social contexts, and increased independence—all of which can intensify feelings of uncertainty or social anxiety, particularly among those with lower baseline social confidence or fewer established support systems (Bewick et al., 2010; Conley et al., 2014). Younger students may therefore be more susceptible to social-cognitive vulnerability during this critical transitional period. 

Within our university population, the two profiles of social functioning were distinguished by different levels of social awareness, social cognition, social communication, social motivation, and restricted and repetitive behaviors/interests. We observed a general trend where the mean profile scores increased consistently across profiles, but social communication and restricted, repetitive behaviors and interests emerged as more distinct characteristics differentiating the two profiles. Given the importance of these traits within ASD, it is not surprising that they played a more significant role in distinguishing the profiles within this university population. Communication deficits, including challenges in social interaction, verbal and nonverbal communication, and maintaining reciprocal conversations (Hodges et al., 2020; Lord et al., 2018), can substantially affect individual functioning in a university setting, where social and communicative interactions are crucial for academic success and social integration (Gurbuz et al., 2018). In addition, restricted, repetitive behaviors and interests include a range of actions, from repetitive motor movements to insistence on sameness and highly focused interests (Tian et al., 2022). In the context of university students, these behaviors may affect daily routines, learning styles, and social interactions, further distinguishing students who experience non-social functioning difficulties. As a result, providing more inclusive activities could help enhance engagement levels and reduce the risk of social isolation (Sosnowy et al., 2017).

### 4.2. Social Functioning Difficulties Impacting Academic Engagement

It is noteworthy that students within the social functioning difficulties profile reported the lowest scores for indicators of academic engagement. This profile was found to be less inclined to pursue further education at university level if they had alternative options available to them. They were more likely to regard university as a valuable opportunity for their personal development and to believe in their ability to persevere in their chosen field of study. They were also more likely to share their professional plans with their family and to recognize the importance of fostering relationships with their university peers. Similarly, McLeod and Anderson (2023) found a relationship between autistic traits behaviors and academic and social college outcomes among university students, regardless of diagnostic status. Regarding academic and social outcomes, they observed consistent associations between autistic traits and lower grade point averages, course failure, and academic difficulties. They also observed that students with higher autistic traits were less likely to have a close confidante, reported lower friendship quality, and experienced social exclusion.

Many authors increasingly acknowledge that, as in any human experience, the role of social relations, contextual frames, and shareable experiences by means of which students interact with and confront each other cannot be ignored in a dynamic conceptualization of engagement (Alivernini et al., 2019; Kahu, 2013; Shernoff et al., 2016). The social dimensions of academic engagement have been shown to facilitate the acknowledgement of its relational aspects, including the sharing of resources and ideas with teaching staff and fellow students, as well as between families (Cavicchiolo et al., 2019a; Girelli et al., 2019). This evidence could be in line with the idea that social functioning with peers can greatly influence student motivation and their ability to adjust to educational environments (Cavicchiolo et al., 2019b).

In this context, engagement is defined by an individual’s capacity to navigate their position within complex social systems involving diverse actors. The ability to flexibly differentiate social relationships is seen as a key characteristic of engaged students. Thus, our study suggests how socially vulnerable university students have a risk of lower academic engagement and less ability to manage difficulties, potentially decreasing their motivation and involvement in learning-related activities, with a lower utilization of the social network needed to implement suitable coping strategies (Abbott-Chapman et al., 2013; Girelli et al., 2018; Klem & Connell, 2004).

Interestingly, individuals with social functioning difficulties do not experience more difficulties with their university professors compared to their peers, as indicated by the regression analysis. Thus, relationships with faculty members play a crucial role not only in enhancing student self-regulation (Williams & Deci, 1996), but also in fostering and developing student intrinsic motivation, which can help prevent dropouts (Girelli et al., 2018; Hardré & Reeve, 2003). Higher education environments, particularly within universities, allow for more individualized interactions between students and professors, which may reduce the social pressures typically encountered in earlier educational stages. Professors may thus serve as a valuable resource, leading to more inclusive communication styles and methods. Additionally, the value they attribute to their program of study for personal professional growth is similar between the two profiles. This similarity suggests that, despite differences in social functioning, students perceive their academic pursuits as equally beneficial. This may indicate that impairments in social functioning do not diminish an individual’s ability to recognize the importance of education for future career development.

### 4.3. Social Functioning and Perception of Emotional Distress and Learning Difficulties

Our observations suggest that difficulties in social functioning can be a risk factor for internalizing problems such as anxiety and depression, mirroring findings in other disorders during adulthood (Iaia et al., 2024; O’Rourke et al., 2020). Specifically, higher levels of anxiety and depression symptoms increase the probability of social impairment by 9% and 19%, respectively. This evidence aligns with recent literature examining the link between autistic traits and social outcomes in neurotypical university student populations (Reed et al., 2016). In a study of college undergraduates, Jobe and White (2007) found a negative correlation between AQ scores and both the number of reported friendships and the duration of the closest friendship. Wainer et al. (2013) showed that higher scores on the Broad Autism Phenotype Questionnaire (BAPQ) were associated with a reduced desire for close relationships, increased loneliness, lower friendship quality, and more negative interactions. Similarly, Stice and Lavner (2019) identified a significant link between BAPQ scores and internalizing symptoms, mediated by social connectedness and loneliness among undergraduates (see also (Faso et al., 2015; Sasson et al., 2012)).

Individuals with social functioning difficulties often struggle in other cognitive domains—including attention, executive functioning, language, and learning (Brimo et al., 2021). Shirama et al. (2022), for example, showed that children with social functioning difficulties performed worse across these domains, and that this poorer performance was linked to greater emotional and behavioral problems. Such findings imply that the cognitive weaknesses accompanying social functioning difficulties can compound both learning and emotional challenges. Consistent with this evidence, we found greater perceived difficulties in different aspects of learning (reading, writing, and calculation), as measured by a self-report questionnaire, in students identified as having social functioning difficulties, suggesting a possible association between social skills and learning difficulties. It should be remembered with respect to this result that the questionnaire used (Vinegrad Plus) does not quantitatively measure the difficulties experienced by these subjects, but rather their awareness that they may have some difficulties in some of these aspects related to the use of formal learning in activities of daily living. This suggest that interventions can be better tailored to support academic adaptation in students, enhancing their learning experiences and promoting their overall success in higher education. The need for appropriate support for individuals with social functioning difficulties in universities is critical. Cage and Howes (2020) highlighted that autistic individuals often experience heightened social problems, which can persist over time. This enduring anxiety can lead to chronic stress, diminished academic performance, and even premature departure from university, largely due to the social demands of higher education (Freeth et al., 2012). Targeted social skill interventions and systematic support services within university settings are essential for supporting students with social functioning difficulties. These measures should not be limited to those with a formal diagnosis, but should also address the needs of students who, despite not meeting diagnostic criteria, experience significant challenges in social communication, interaction, or adaptation. Adopting such an inclusive approach would acknowledge the continuum of social-cognitive vulnerabilities and ensure that support strategies are responsive to functional needs rather than categorical labels (Happé et al., 2016).

### 4.4. Limitations and Strengths

This study is not without limitations. First, the number of male participants was relatively small. A larger sample would have allowed sex-stratification analyses. Because males and females encounter distinct biological and social risks and protective factors across their lifespan, examining these associations in women is clinically important. The skewed sex distribution likely reflects our recruitment from education courses, which tend to enroll more females. This imbalance may affect the generalizability of our findings and limit our ability to draw conclusions about the experiences and characteristics of males with social communication vulnerability. Nevertheless, there is little known about females, and our sample size was large enough to use rigorous statistical methods and to take effect sizes into account for each comparison (Footitt et al., 2024; Kongsted & Nielsen, 2017).

Second, while we employed an assessment tool originally developed for individuals with ASD, it is important to acknowledge that the identified social functioning difficulties profiles may also encompass individuals exhibiting limited social and communication skills due to a variety of other conditions. These could include, for example, non-pathological factors such as mild levels of social anxiety, limited social exposure, or individual personality traits that may affect social interactions and communication skills. Therefore, the social functioning profile described in this study should not be interpreted as exclusively indicative of autistic traits, but rather as a broader indicator of social functioning challenges.

In addition, given the cross-sectional design, the directionality of the relationships between social functioning and academic engagement or emotional distress remains uncertain, and reciprocal influences between these variables cannot be excluded.

In terms of strengths, this investigation addresses a critical need for population-based assessments, particularly using clinician-administered tools. A key strength of this study lies in its population-based approach to assessing social functioning difficulties using validated, clinician-developed tools. By excluding participants with confirmed diagnoses of autism spectrum disorder or other neurodevelopmental conditions (Kim et al., 2015; Préfontaine et al., 2022), we focused on identifying meaningful individual differences in social functioning within a non-clinical university population. This design allowed us to make a nuanced distinction between stable personality traits and more functionally impactful social-cognitive vulnerabilities. While some individuals may exhibit persistent difficulties in social interaction, communication, or adaptability, these challenges do not necessarily reflect a diagnosable condition, but may still significantly influence academic engagement and well-being.

Social functioning difficulties—particularly within the demanding environment of higher education—may indicate a need for support, even in the absence of clinical symptoms. By identifying subgroups of students at risk for reduced academic engagement and poorer adaptation to university life, this study highlights the importance of functional, rather than diagnostic, perspectives. These findings support the development of inclusive interventions tailored to the needs of students with varying social capacities, helping ensure broader access to effective academic and psychosocial support.

## 5. Conclusions

In conclusion, this study explored the mechanisms that can affect student involvement and psychological well-being. Difficulties in social communication and interaction may reduce student motivation and involvement in academic activities, increase the risk of chronic psychological stress, lower academic performance, and potentially influence a premature departure from university.

In terms of clinical policies, universities can take several steps to support students with social functioning difficulties and improve their academic engagement and well-being. First, implementing an initial screening process for incoming students could help identify those with social skill deficit vulnerability and/or learning difficulties, as well as psychological distress. This would enable institutions to provide tailored support services early on, addressing potential challenges before they escalate. Further, creating more inclusive environments with targeted programs and activities that encourage social integration can provide a stronger sense of belonging for students who may struggle with social interactions. This could include mentorship programs, peer support groups, or workshops that promote social skills development. Universities can also provide tailored services that address the specific needs of students with social functioning difficulties, offering accessible counseling services, stress management programs, and mental health resources. These services should aim to proactively address individual behaviors that can optimize social functioning. This could involve personalized academic advice, flexible course selection, and accommodation for those struggling to engage with peers or professors in traditional settings. Further, faculty members should focus on enhancing both the quantity and quality of social support, while encouraging meaningful student interactions. By establishing a supportive social and academic framework, universities can help vulnerable students remain engaged, persist in their studies, and develop effective coping strategies, ultimately aiding them in achieving their academic aspirations.

## Figures and Tables

**Table 1:** Properties of the two latent profiles according to sex and age.

		Profile 1	Profile 2	Total
		N = 311	N = 229	
Sex	Male (%)	29 (9.5%)	25 (11.1%)	54 (10.2%)
	Female (%)	274 (89.5%%)	197 (87.6%)	471 (88.7)
	Missing (%)	-	-	15 (1.1%)
Mean age (SD)		23 (6.06)	21.7 (3.45)	

Note. Profile 1: “Non-social functioning difficulties”; Profile 2: “Social functioning difficulties”.

**Table 2:** Comparison of SRS-2, SAES, Vinegard Plus, GAD-7, and PHQ-9 among the two identified latent profiles (N = 540).

Variable	Profile 1	Profile 2	F	df1, df2	*p*	η^2^p
	*Mean* (*SD*)	*Mean* (*SD*)				
Awareness	58.1 (6.56)	61.7 (8.70)	27.70	1407	0.001	0.06
Cognition	53.3 (6.70)	61.7 (8.32)	160.30	1426	0.001	0.27
Communication	50.6 (5.47)	66.5 (9.51)	510.90	1338	0.001	0.60
Motivation	54.5 (6.35)	65.7 (8.55)	277.30	1402	0.001	0.41
Restricted Interests and Repetitive Behavior	53.1 (3.29)	60.0 (6.95)	191.30	1304	0.001	0.39
SAES—Perceived ability to persist in their choice to attend university	17.5 (2.64)	15.82 (3.44)	38.40	1412	0.001	0.08
SAES—Sense of university value and belonging	25.85 (3.47)	24.93 (4.16)	7.35	1437	0.007	0.02
SAES—Value of university program	30.06 (4.31)	28.55 (5.19)	12.81	1436	0.001	0.03
SAES—Relationships between university and personal network	11.54 (2.42)	28.55 (5.19)	33.36	1426	0.001	0.07
SAES—Engagement with university peers	18.85 (3.68)	17.31 (4.65)	17.32	1421	0.001	0.04
SAES—Engagement with university professors	13.60 (2.93)	12.75 (3.08)	10.39	1477	0.001	0.02
Vinegrad Plus	1.55 (1.28)	2.44 (1.68)	44.46	1410	0.001	0.10
Anxiety	7.81 (3.94)	11.53 (4.49)	99.99	1453	0.001	0.18
Depression	6.53 (3.50)	10.88 (5.38)	114.40	1366	0.001	0.24

Note. Profile 1: “Non-social functioning difficulties”; Profile 2: “Social functioning difficulties”. η^2^p = Partial Eta Squared.

**Table 3:** Hierarchical logistic regression model predicting age, sex, learning difficulties, psychological distress, and academic engagement for the two latent profiles.

Predictor	Exp(*B*)	95% CI for Exp(*B*)	*B*	SE	*z*	*p*
Block 1						
Age	0.957	[0.922, 0.992]	−0.0443	0.0186	−2.383	0.017 *
Sex	0.868	[0.508, 1.482]	−0.1419	0.2730	−0.520	0.603
Block 2						
Anxiety	1.090	[1.026, 1.16]	0.0863	0.0310	2.79	0.005 *
Depression	1.191	[1.115, 1.27]	0.1744	0.0330	5.28	0.001 *
Block 3						
Vinegrad Plus	1.149	[1.079, 1.222]	0.1385	0.0318	4.353	0.001 *
SAES—Perceived ability to persist in their choice to attend university	0.902	[0.836, 0.972]	−0.1036	0.0385	−2.689	0.007 *
SAES—Sense of university value and belonging	1.096	[1.003, 1.197]	0.0917	0.0451	2.032	0.042 *
SAES—Value of university program	0.976	[0.915, 1.040]	−0.0247	0.0326	−0.756	0.450
SAES—Relationships between university and personal network	0.867	[0.793, 0.949]	−0.1423	0.0458	−3.106	0.002 *
SAES—Engagement with university peers	0.940	[0.888, 0.994]	−0.0623	0.0290	−2.151	0.031 *
SAES—Engagement with university professors	0.939	[0.870, 1.015]	−0.0625	0.0393	−1.592	0.111

Note. CI = confidence interval. The reference category for the dependent variable was “Profile 1: Non-social functioning difficulties”. Exp(*B*) = odds ratio. * *p* < 0.05.

## Data Availability

Data are available upon request from the authors.

## Appendix A. Latent Profile Identification

Table A1 reports our initial testing of sixteen potential model comparisons, which included adjustments to the number of classes and parameterization of variance–covariance structures, starting from the most (model 1) to the least restrictive (model 6). Based on the current results, we focused on model 6 (Class-varying unrestricted parameterization). This model was further examined because it displayed a lower BIC value.

**Table A1:** Sixteen potential model comparisons, which include adjustments to the number of classes and parameterization of variance–covariance structures.

Model	Classes	AIC	BIC	AWE	CLC	KIC
1	1	19,136.033	19,178.948	19,269.864	19,118.033	19,149.033
1	2	18,516.933	18,585.598	18,732.592	18,486.605	18,535.933
1	3	18,354.734	18,449.149	18,651.996	18,312.301	18,379.734
1	4	18,255.995	18,376.158	18,634.738	18,201.579	18,286.995
2	1	19,136.033	19,178.948	19,269.864	19,118.033	19,149.033
2	2	18,420.479	18,510.602	18,704.146	18,380.058	18,444.479
2	3	18,241.502	18,378.832	18,674.639	18,179.025	18,276.502
2	4	18,155.715	18,340.253	18,738.228	18,071.278	18,201.715
3	1	18,262.623	18,348.455	18,532.286	18,224.623	18,285.623
3	2	18,194.366	18,305.947	18,545.899	18,143.994	18,223.366
3	3	18,206.431	18,343.761	18,640.286	18,143.236	18,241.431
3	4	18,216.912	18,379.991	18,732.162	18,141.821	18,257.912
6	1	18,262.623	18,348.455	18,532.286	18,224.623	18,285.623
6	2	18,121.531	**18,297.485**	18,677.328	18,040.643	18,165.531
6	3	18,094.825	18,360.903	18,935.799	17,972.006	18,159.825
6	4	18,108.213	18,464.413	19,234.278	17,943.549	18,194.213

Note. This table compares different profile numbers for four potential combinations of model parameters, including equal/fixed classes and equal/equal covariances. Results are highlighted in bold to indicate the optimal model parameterization according to the best information criterion. The information criteria used included AIC (Akaike Information Criterion), BIC (Bayesian Information Criterion), AWE (approximate Weight of Evidence Criterion), CLC (classification Likelihood Criterion), and KIC (Kullback Information Criterion).

Table A2 documents the results of further testing aimed to improve the fit indices for the models across the two selected profiles. Model 6 with two profiles generated better AIC and BIC values. Additionally, model 6 demonstrated good classification accuracy (entropy = 0.58) and a significant BLRT-p value. Therefore, we opted for model 6 with two profiles, due to its optimal fit among the tested models. The entropy values for the proposed model 6 were satisfactory (0.58). We characterized the two latent profiles as “Non-social functioning difficulties” (Profile 1) and “Social functioning difficulties” (Profile 2). The proportion of participants in each estimated profile was n = 311 (57.6%) for Profile 1, and n = 229 (42.41%) for Profile 2. It should be noted that the smallest class proportion and number exceeded 5% and/or N > 50, as recommended by O’Donnell et al. (2017).

**Table A2:** Fit indices for the models across the two selected profiles.

Model	Classes	AIC	BIC	Entropy	N_min	**BLRT_p**
6	1	18,263.39	18,349.22	1.00	1.00	
**6**	**2**	**18,124.95**	**18,300.90**	**0.58**	**0.50**	**0.01**
6	3	18,125.95	18,392.03	0.69	0.16	0.02
6	4	18,122.86	18,479.06	0.72	0.11	0.02

Note. The current table indicates that the model with two profiles exhibited lower BIC values and a good entropy value, resulting in improved differentiation between profiles. The information criteria used were AIC (Akaike Information Criterion) and BIC (Bayesian Information Criterion). N_min (Proportion of the sample assigned to the smallest class). The BLRT_p(Bootstrapped Likelihood Ratio Test) was employed for comparison. Values highlighted in bold denote the optimal model based on the criteria used.
